# Using Stochastic modelling to identify unusual continuous glucose monitor measurements and behaviour, in newborn infants

**DOI:** 10.1186/1475-925X-11-45

**Published:** 2012-08-06

**Authors:** Matthew Signal, Aaron Le Compte, Deborah L Harris, Phil J Weston, Jane E Harding, J Geoffrey Chase

**Affiliations:** 1 BE(Hons), Department of Mechanical Engineering, University of Canterbury, Christchurch, New Zealand; 2 Department of Mechanical Engineering, University of Canterbury, Christchurch, New Zealand; 3 Liggins Institute, University of Auckland, Private Bag 92019, Auckland, New Zealand; 4 Newborn Intensive Care Unit, Waikato District Health Board, Private Bag 3200, Hamilton, New Zealand

**Keywords:** Continuous Glucose Monitor, Classify, Neonatal, Intensive care unit, Glycaemia

## Abstract

**Background:**

Abnormal blood glucose (BG) concentrations have been associated with increased morbidity and mortality in both critically ill adults and infants. Furthermore, hypoglycaemia and glycaemic variability have both been independently linked to mortality in these patients. Continuous Glucose Monitoring (CGM) devices have the potential to improve detection and diagnosis of these glycaemic abnormalities. However, sensor noise is a trade-off of the high measurement rate and must be managed effectively if CGMs are going to be used to monitor, diagnose and potentially help treat glycaemic abnormalities.

**Aim:**

To develop a tool that will aid clinicians in identifying unusual CGM behaviour and highlight CGM data that potentially need to be interpreted with care.

**Methods:**

CGM data and BG measurements from 50 infants at risk of hypoglycaemia were used. Unusual CGM measurements were classified using a stochastic model based on the kernel density method and historical CGM measurements from the cohort. CGM traces were colour coded with very unusual measurements coloured red, highlighting areas to be interpreted with care. A 5-fold validation of the model was Monte Carlo simulated 25 times to ensure an adequate model fit.

**Results:**

The stochastic model was generated using ~67,000 CGM measurements, spread across the glycaemic range ~2-10 mmol/L. A 5-fold validation showed a good model fit: the model 80% confidence interval (CI) captured 83% of clinical CGM data, the model 90% CI captured 91% of clinical CGM data, and the model 99% CI captured 99% of clinical CGM data. Three patient examples show the stochastic classification method in use with 1) A stable, low variability patient which shows no unusual CGM measurements, 2) A patient with a very sudden, short hypoglycaemic event (classified as unusual), and, 3) A patient with very high, potentially un-physiological, glycaemic variability after day 3 of monitoring (classified as very unusual).

**Conclusions:**

This study has produced a stochastic model and classification method capable of highlighting unusual CGM behaviour. This method has the potential to classify important glycaemic events (e.g. hypoglycaemia) as true clinical events or sensor noise, and to help identify possible sensor degradation. Colour coded CGM traces convey the information quickly and efficiently, while remaining computationally light enough to be used retrospectively or in real-time.

## Background

Abnormal blood glucose (BG) concentrations have been associated with increased morbidity and mortality in both critically ill adults and infants. Patients in the intensive care unit (ICU) often experience high levels of insulin resistance [1-7] and stress-induced hyperglycaemia, which can negatively impact outcomes [1-3,7,8]. Similarly, hyperglycaemia in very-low-birth-weight or premature infants is also associated with negative outcomes [9,10]. Further complicating the matter, hypoglycaemia and glycaemic variability have both been independently linked to mortality in critically ill patients [11-14].

Diagnosis of hyperglycaemia and hypoglycaemia in critically ill patients is by BG measurements, which are typically taken several hours apart. More frequent BG measurements are not clinically practical due to the additional nursing workload [15-17] and in preterm infants, blood volume considerations. Consequently, important glycaemic events between BG measurements can go undetected. Continuous Glucose Monitoring (CGM) devices have the potential to improve the detection and diagnosis of these glycaemic abnormalities. The continuous glucose monitoring system (CGMS® System Gold™ Medtronic, Minimed, Northridge, CA, USA) provides a glucose value every 5 minutes or 288 measurements per day, with only 2–4 BG measurements per day required for device calibration.

There have been relatively few successful investigations of CGMs in critical care use [18-21], although they are well studied in Type 1 diabetes [22-24]. In particular, one set of tight glycaemic control trials using CGM technology was not particularly successful due, in part, to significant sensor noise [25,26]. In some cases, added sensor noise is a trade-off for the CGM’s far higher, automated sampling rate [18,27] and must be effectively managed for these devices to be used successfully. However, these sensor and algorithm technologies are also constantly evolving with every new generation offering improvements [24,28].

If CGM devices are to be used in the clinical setting to monitor, diagnose and potentially aid in the treatment of abnormal glycaemia, clinicians need to know the data are reliable and accurate. Consider a scenario in which CGM data are retrospectively analysed to classify hypoglycaemia in neonates, where frequent BG measurements are not available. Three consecutive measurements in a CGM trace read 4 mmol/L, 2.5 mmol/L, followed by 4 mmol/L. If hypoglycaemia was classified as a measurement below 2.6 mmol/L, then this would be recorded as a hypoglycaemic event. However, if the rest of the CGM trace was very stable with low variability, intuition would suggest this 'event' is potentially a sensor artefact. There currently is no reliable method to determine which of these interpretations is more likely to be “true”.

Our manuscript describes a tool that will aid clinicians in identifying unusual CGM behaviour, retrospectively or in real-time, and highlight sections of the CGM glucose trace that potentially need to be interpreted with care. Specifically, the focus is to identify unusual CGM sensor behaviour, not unusual glycaemic excursions.

## Subjects and methods

### Subjects

This study used CGM data from 50 babies at risk of hypoglycaemia who were admitted to the Waikato Hospital Newborn Intensive Care Unit (NICU). The cohort contained 26 males and 24 females with a median gestational age of 34 weeks and a median birth weight of 2172 g. The primary risk factors used to identify infants likely to become hypoglycaemic include diabetic mother, prematurity and being small or large for gestational age. The study was approved by the Northern Y Regional Ethics Committee.

### Continuous glucose monitoring

All patients had interstitial glucose monitoring using the CGMS® System Gold™ (Medtronic, Minimed, Northridge, CA, USA). Monitoring began on admission to the NICU and finished after 7 days or when the baby was no longer considered to be at risk of hypoglycaemia, whichever came first. During the monitoring period nurses were asked to record all BG concentrations, feeding and medication for the management of hypoglycaemia. However, they remained blind to the glucose concentrations determined by the device. The device was calibrated per the manufacturer’s recommendations and all of the data entered into the device were checked against clinical records for accuracy. Upon completion of monitoring, data were downloaded to a PC using CGMS system solutions software version 3.0C, which calibrated the CGM readings retrospectively. A total of 234 days of CGM data (67438 measurements) were collected and the per-patient median [IQR (inter-quartile range)] duration of monitoring was 4.7 [4.0 - 5.7] days.

### Calibration measurements

The CGM device required calibration 2–4 times daily to convert the electrical current produced by the sensor into a meaningful glucose value. Blood samples were taken by nursing staff via heel-pricking and the glucose concentration was used to calibrate the CGM device. The median [inter-quartile range (IQR)] interval between samples was 4.8 [3.5 – 6.4] hours.

All BG calibration measurements were made using a blood gas analyser (Radiometer, ABL800Flex, Copenhagen) using the glucose oxidase method. This device has a reading range of 0.0 to 60.0 mmol/L and a C.V. of 1.4-2.2% [29,30]. Furthermore, a study by Watkinson et al. showed that a device from the same family, using the same glucose electrode, had a coefficient of variation of 2.1% in ICU patients and performance was not affected by haematocrit, pH or PaO2 [30]. Due to the location of the blood gas analyser, a short time delay (estimated < 15mins maximum) was possible between taking the blood sample and introducing the resulting measurement into the device.

### Stochastic model

A stochastic model based on the kernel density method was implemented to classify unusual CGM measurements using the previous CGM measurement and information about the history of CGM behaviour. The model is an extension to the methods described by Lin et al. [27] who developed a stochastic model for insulin sensitivity prediction.

(1) $$P(CGM_{n}=y|CGM_{n-1}=x)=\sum_{i=1}^{n}\omega_{i}\left(x\right)\frac{\phi \left(y;y_{i},\sigma_{y_{i}}{}^{2}\right)}{p_{y_{i}}}$$

(2) $$\omega_{i}\left(x\right)=\frac{\phi \left(x;x_{i},\sigma_{x}{{}_{{}_{i}}}^{2}\right)/p_{x}{}_{{}_{i}}}{\sum_{j}^{n}{=}_{1}\phi \left(x;x_{j},\sigma_{x_{j}}{}^{2}\right)/p_{x_{j}}}$$

Where: $p_{x_{i}}=\int_{0}^{\infty }\phi \left(x;x_{i},\sigma_{x_{i}}{}^{2}\right)$ and $p_{y_{i}}=\int_{0}^{\infty }\phi \left(y;y_{i},\sigma_{y_{i}}{}^{2}\right)$

The model was generated using Equations 1 and 2 which define the 2-dimensional kernel density estimation in conditional CGM measurement variability. Each $\phi \left(x;x_{i},\sigma_{x_{i}}{}^{2}\right)$ and $\phi \left(y;y_{i},\sigma_{y_{i}}{}^{2}\right)$ is a normal probability density function (pdf) centred at the corresponding x_i_ and y_i_. To force non-negativity in x and y, $p_{x_{i}}$ and $p_{y_{i}}$ normalise each $\phi \left(x;x_{i},\sigma_{x_{i}}{}^{2}\right)$ and $\phi \left(y;y_{i},\sigma_{y_{i}}{}^{2}\right)$ in the positive domain. Specifically, for a given CGM measurement *CGM*_*n-1*_, Equations 1 provides a continuous, empirical estimate of the conditional pdf for the next CGM measurement, *CGM*_*n*_. These conditional pdf’s provide the basis for classifying CGM measurements and identifying unusual CGM behaviour.

### CGM measurement classification

Using the stochastic model, a given CGM measurement, *CGM*_*n*_, is classified as follows:

1. The previous measurement, *CGM*_*n-1*_, is used to find the corresponding conditional pdf from the model.

2. *CGM*_*n*_ is located in the pdf and its percentile value in the conditional pdf is determined.

3. The percentile is used to classify *CGM*_*n*_, where a very high or very low percentile is indicative of an outlier. These outliers are classified as unusual CGM measurements.

The measurement-to-measurement sections of the CGM trace were colour coded based on the percentile value, to highlight areas of unusual CGM behaviour quickly and effectively. Three confidence intervals (CI’s) were used to specify the colour: within 80% CI (10th-90th percentile) was blue, within 90% CI (5th-95th percentile) was cyan, within 99% CI (0.5th-99.5th percentile) was yellow, and outside 99% CI was red. These intervals were chosen based on the data used in this study and can be customised for different patient groups and/or different CGM sensors. As the scale starts at 80% CI, the focus here is on classifying outliers, rather than the full range.

### 5-fold validation of stochastic model

A 5-fold validation was used to check the fit of the stochastic model. The data set (N = 50) was randomly divided into 5 sets of 10 patients. For each 10 patient group, the remaining 40 patients were used to create a stochastic model which was then tested on the group of 10 patients. The model fit was assessed by counting the number of clinical CGM measurements (from the 10 patients) captured by the model’s 80% CI, 90% CI and 99% CI.

Due to the random nature of this validation procedure, Monte Carlo (MC) methods were used to reduce the effect of randomly selected outliers on overall results. MC methods provide a robust means of estimating the range of possible outcomes for a process involving one or more random variables. In this study, the MC simulation involved repeating the 5-fold validation 25 times, and reporting the Median [IQR] results.

## Results

### Clinical CGM data and stochastic model generation

**Figure 1:**
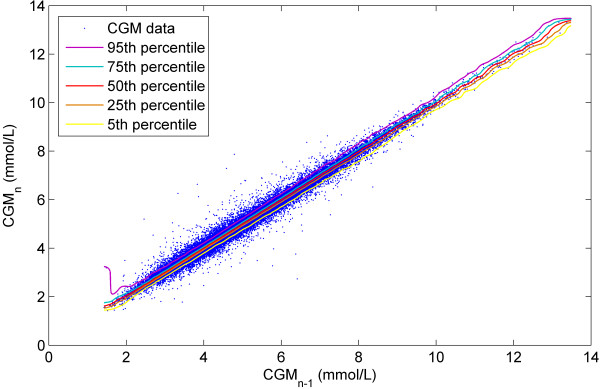
**CGM**_**n-1**_**vs. CGM**_**n.**_ Plot of CGM measurement pairs (CGM_n-1_, CGM_n_) with contour lines representing the 5th to 95th percentiles, from the bottom of the plot up.

**Figure 2:**
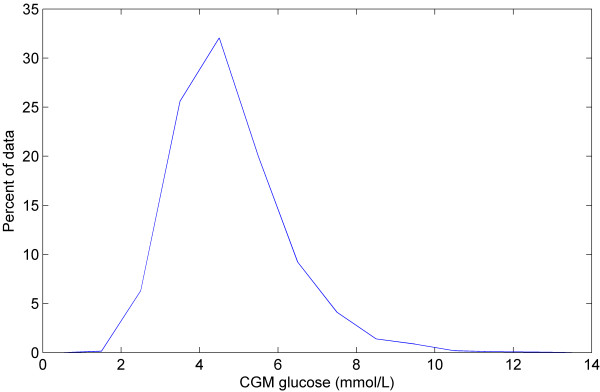
**Data density by glycaemic level.** Density of the data set by glycaemic level. Density is shown as a percent of the total data set (67,438 measurements).

**Figure 3:**
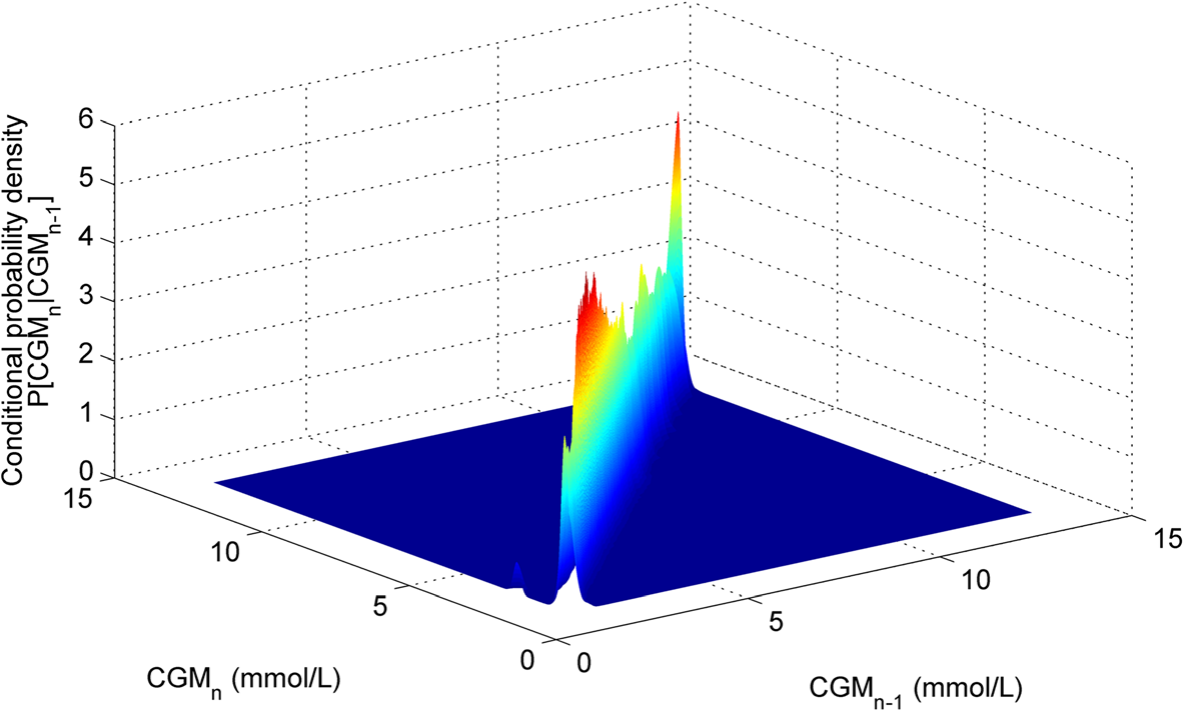
**Stochastic model surface.** Data density by glycaemic level. Stochastic model surface for this data set. Conditional probability density functions are the surface slices along CGM_n-1_axis, each slice has an area under the curve summing to 1.0. A colour gradient was used to show the height of the surface.

**Figure 4:**
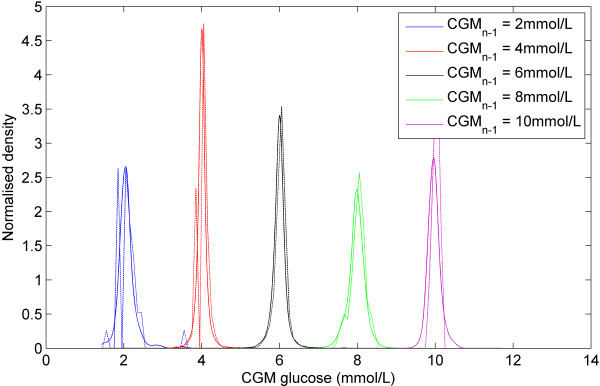
**Model surface vs. clinical data.** Comparison of conditional probability density functions at different CGM_n-1_. Pdf's from the model are solid lines and empirical pdf's from actual CGM data are dotted.

Figure 1 shows a plot of all the CGM data (*CGM*_*n-1*_, *CGM*_*n*_). The contour lines represent the 5th, 25th, 50th, 75th, and 95th percentiles of the stochastic model surface. Figure 2 shows a distribution of the data density by glycaemic level. Figure 3 shows a surface plot of the stochastic model. Conditional probability density functions are slices parallel to the *CGM*_*n*_ axis, and each slice has an area under the curve of 1.0. Figure 4 shows a comparison of the pdf's obtained from the model versus the pdf's obtained directly from the CGM data. Each pdf shows the expected distribution of *CGM*_*n*_ given a previous measurement (*CGM*_*n-1*_) of 2, 4, 6, 8 or 10 mmol/L. It should be noted that the pdf's could be generated for any value of *CGM*_*n-1*_ within the bounds of the model; Figure 4 shows just five examples.

### Classification of representative CGM data

**Figure 5:**
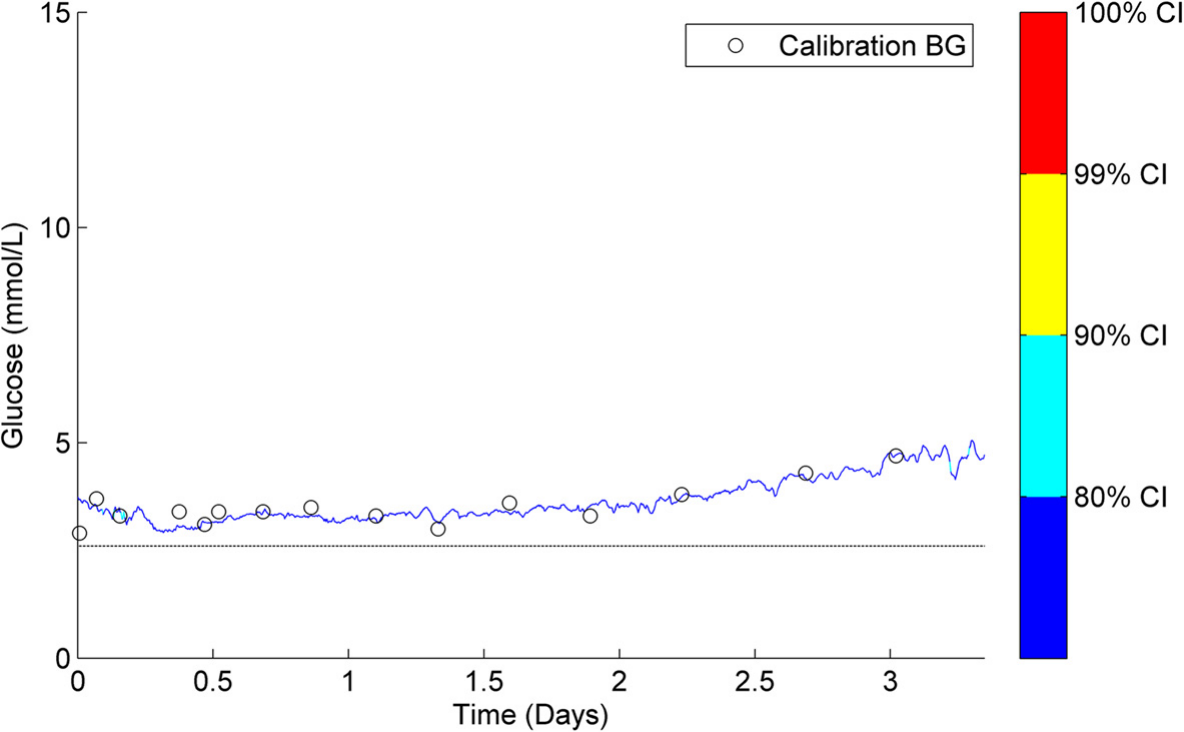
**Classification example 1.** Stable CGM trace with no yellow or red measurements indicating no CGM measurements were classified unusual.

**Figure 6:**
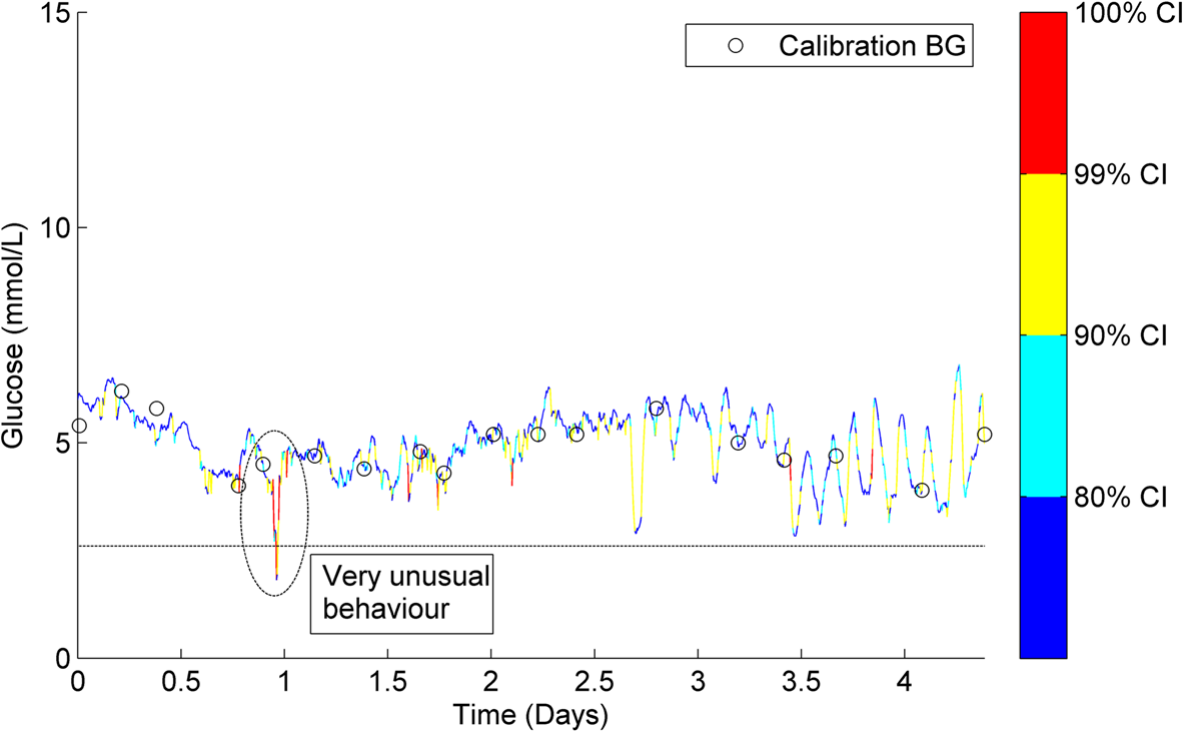
**Classification example 2.** CGM trace with several measurements classified as mildly unusual. Note the hypoglycaemic event at ~1 day which has been classified as very unusual (red).

**Figure 7:**
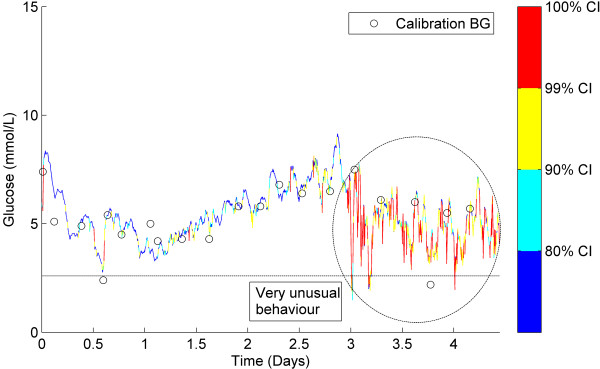
**Classification example 3.** CGM trace with several measurements classified as mildly unusual. After day 3 the trace is classified as very unusual (red) and could be indicative of sensor malfunction.

Figures 5, 6, 7 show three examples of CGM traces that have been coloured using the stochastic classification method. Figure 5 shows a stable trace, which is almost entirely dark blue, indicating the measurement-to-measurement change throughout the trace is not unusual. Figure 6 shows a trace with several potentially unusual measurements throughout the trace. The hypoglycaemic event that occurs at approximately one day after monitoring began is coloured red and classified as very unusual. Figure 7 shows a trace with a few potentially unusual measurements for the first three days of monitoring. After day 3 a high proportion of the CGM measurements are classified as very unusual and are coloured red.

## Discussion

### Clinical CGM data and stochastic model generation

The aim of this study was to design a tool that could aid clinicians in identifying unusual CGM behaviour that should potentially be interpreted with care. Stochastic modelling methods from [31] and a method of colouring CGM traces were used to highlight unusual CGM behaviour clearly and efficiently, in either real-time or retrospectively.

Figures 1 and 2 give information about the raw data used to create the stochastic model. More than 99% of the data is within 2-10 mmol/L range, shown in Figure 2. There are several outliers in Figure 1 that have a very large change in glycaemia over the 5 minute measurement interval. The high data density means these outliers have little effect on the model fit, shown by the smooth and tight percentile lines in Figure 1. However, below 2 mmol/L there are 97 CGM measurements and due to the relatively low density of data the outliers have more impact on the model fit. This effect is clearly seen in the 95th percentile line of the model, which strays upward at levels below 2 mmol/L. Similarly, above 10 mmol/L there are only 232 measurements and the percentile lines all have a wave-like shape, again showing the effect of outliers where data density is low. A greater data density would alleviate these issues without changing the approach in this proof-of-concept.

The quality of data used to create a stochastic model will affect how future CGM measurements are classified. If a model is created using low quality data, containing a significant number of outliers, then the model could potentially classify future outlier measurements as ‘usual’ or ‘expected’. Data quality is particularly important when using small data sets, as single outliers can have more effect on the overall model. However, the growing use of databases and electronic records means that collecting large amounts of data that are fully representative will become easier, even for sub-cohorts. Thus, this work foreshadows an application with potential for good clinical use in future, as the method of creating such models is general.

Figure 3 shows the surface of the stochastic model. The colour gradient shows how the shape of the model changes in the domain of *CGM*_*n-1*_ and that a single, global probability density function is not applicable to this data set. Figure 4 further reinforces this with 5 pdf’s taken from the model at different *CGM*_*n-1*_ values, resulting in 5 different shaped density functions. These pdf’s are also used to show that the model fits the empirical data well. The model pdf’s (solid lines) overlay the empirical data (dotted lines) with only minor discrepancies.

Additionally, the model fit was checked using a 5-fold validation, with results shown in Table 1. Across the 25 MC runs, the 80% CI captured 83% of the data (median), the 90% CI captured 91% of the data (median) and 99% CI captured 99% of the data (median). These results show the model to capture the CGM data well over the expected range of CGM values, with little variation in the percentage of data captured in each CI.

**Table 1:** Results from a 5-fold validation of the model

5-fold Model Validation (25 MC runs)	80% CI	90% CI	99% CI
Variation across MC runs (Median [IQR])	0.83 [0.79 - 0.86]	0.91 [0.89 - 0.93]	0.99 [0.98 - 0.99]

### Classification of representative CGM data

Figures 5, 6, 7 show 3 different CGM data sets and how the stochastic model classified the individual CGM measurements within them. Figure 5 shows a very stable, flat CGM trace with only small variations over the 3.5 days of monitoring. The CGM trace passes near all calibration measurements and there doesn’t appear to be any unusual CGM behaviour. The stochastic model classified almost the entire trace as dark blue indicating no unusual CGM behaviour. The interpretation of this trace would not likely be influenced with the additional information provided by the model.

Figure 6 shows a less stable CGM trace with a lot more variability. This trace contains a few yellow and red sections that potentially need to be interpreted with care. The focus of this discussion is the ‘hypoglycaemic event’ that occurs at ~ day 1. In the sequence of 5 measurements that lead up to the 1.8 mmol/L minimum, there are two drops of ~1 mmol/L per 5 minute measurement interval. The model has determined these are extreme outliers and consequently they have been coloured red. The trace then rises to above 4 mmol/L in 5 measurements, similarly with two rises of ~1 mmol/L per 5 minute measurement interval. Although the physiological limits of glucose rate-of-change are still unknown, the level of sensor error that has been reported in previous CGM studies [18,22] suggest that this hypoglycaemic event could potentially be either glycaemia or sensor error.

Interestingly, the bottom of the ‘hypoglycaemic event’ at ~ day 1 contains a dark blue classification; indicating the measurement-to-measurement change immediately after the nadir was not unusual. This was not unexpected, because the lag 1 model classified the change between any two consecutive CGM measurements independent of previous classifications. Additionally, the data in Figure 1 is generally centred on the line *CGM*_*n-1*_ = *CGM*_*n*_ and consequently the model surface in Figure 3 peaks along or near this line. Thus, for the hypoglycaemic event at day 1 when the CGM reported 1.8 mmol/L followed by 1.9 mmol/L, *CGM*_*n-1*_ ≈ *CGM*_*n*_, and the change was classified as expected (blue – within 80% CI).

It is important to note that the aim of the stochastic model is not to try and determine the accuracy of the CGM device or the specific cause of a drop in CGM glucose, but rather to highlight the fact it should be interpreted with care. Furthermore, if the stochastic model was implemented in a real-time clinical setting and the downward CGM measurements were observed, it would be beneficial for the clinician to know whether the sequence of measurements is typical of CGM devices and that patient cohort. It should also be noted that without an accurate BG measurement at ~1 day, no exact conclusion can be drawn about the whether the hypoglycaemic event in this data was a sensor artefact, or a true glycaemic event.

However, this lack of confirmation is often the reality with CGMs. Clinical protocols might use stochastic information to justify an added BG measurement to clarify a potentially significant event. After an event, such traces would yield insight not present at the bedside.

Figure 7 shows an example of CGM data that becomes increasingly more variable and unstable at approximately day 3 of monitoring. Before day 3, the CGM trace is predominantly blue and cyan with only small patches of yellow and occasionally red. However, after day 3 the CGM trace is almost entirely red indicating the stochastic model has classified these measurements as very unusual. The sudden apparent degradation of reliable CGM measurements could be due to a sensor failure. This is not an unreasonable hypothesis, given the sensors used in this study were validated for 3 days of continuous monitoring. Again, without more frequent, accurate BG measurements during the period after day 3 no definitive conclusions can be drawn. However, this example represents another potential use of this stochastic model classification method that might be useful to users of CGM devices.

Finally, the stochastic model and classification methods were used retrospectively in this study. For real-time use, a stochastic model would be generated using prior CGM data, and CGM measurements would be entered into the system in real-time. Classification of paired CGM measurements (*CGM*_*n-1*_, *CGM*_*n*_) takes a fraction of a second, so the corresponding colour coded segment of CGM trace would be displayed without significant delay (estimated less than 1 second). The major limitation in implementing the method in real-time is the ability to stream CGM data to a computer in real-time. Although the technology is available, no ‘off the shelf’ CGM devices currently offer this feature.

## Conclusions

A stochastic model was shown to be capable of classifying CGM measurements to highlight unusual CGM behaviour. The method uses a colour coded CGM trace to convey the information quickly and efficiently and it is computationally light enough to be used retrospectively or in real-time.

There are several potential uses for the stochastic classification which include, but are not limited to, classification of hypoglycaemia and detection of potential sensor failure. Equally, they can augment alarm methods or be used to more optimally time BG measurements, such as in neonates where blood draws are restricted. Overall, while BG measurements are required to draw definitive conclusions about glycaemic events, the stochastic model provides another level of information to aid users in interpretation and decision making.

## Competing interest

The authors declare that they have no competing interests.

## Authors’ contributions

MS: Obtained main results and wrote the main body of the article, ALC: Assisted in obtaining results and writing, DLH: Collected clinical data and assisted in writing, PJW: Collected clinical data and assisted in writing, JEH: Collected clinical data and assisted in writing, JGC: Assisted in obtaining results and writing. All authors read and approved the final manuscript.

## Financial support

UC Department of Mechanical Engineering, New Zealand, Health Research Council of New Zealand. The project described was supported in part by Grant Number RO1HD069622 from the Eunice Kennedy Shriver National Institute of Child Health & Human Development. The content is solely the responsibility of the authors and does not necessarily represent the official views of the Eunice Kennedy Shriver National Institute of Child Health & Human Development or the National Institutes of Health.
